# The seroprevalence of human papillomavirus by immune status and by ethnicity in London

**DOI:** 10.1186/1750-9378-4-14

**Published:** 2009-09-14

**Authors:** Delphine Casabonne, Tim Waterboer, Kristina M Michael, Michael Pawlita, Liza Mitchell, Robert Newton, Catherine Harwood, Charlotte Proby

**Affiliations:** 1 Cancer Epidemiology Unit, Richard Doll Building, University of Oxford, Old Road Campus, Roosevelt Drive, Headington, Oxford, OX3 7LF, UK; 2 Infection and Cancer Program (F020), German Cancer Research Center (DKFZ), Im Neuenheimer Feld 280, 69120 Heidelberg, Germany; 3 Centre for Cutaneous Research, Institute of Cell and Molecular Science, Barts and the London School of Medicine and Dentistry, Queen Mary, University of London, London E1 2AT, UK; 4 Epidemiology & Genetics Unit, Department of Health Sciences, University of York, Area 3, Seebohm Rowntree Building, Heslington, York, YO10 5DD, UK; 5 Division of Surgery and Oncology, Ninewells Hospital & Medical School, University of Dundee, Dundee DD1 9SY, UK

## Abstract

**Background:**

The natural history of cutaneous HPV is unclear and in particular, seroprevalence among individuals with different levels of immune function and ethnicity is unknown. As part of a study of cutaneous squamous cell carcinoma (SCC) and HPV among organ transplant recipients (OTR) from London, we investigated the seroprevalence and risk factors for 34 HPV types (detected using Luminex technology) among 409 OTR patients without skin cancer (243 Caucasians and 166 non-Caucasians), 367 individuals with end stage renal failure on dialysis (222 Caucasians and 145 non-Caucasians) and 152 immunocompetent (IC) individuals without skin cancer (102 Caucasians and 50 non-Caucasians) to compare the HPV seroprevalence in patients with differing immune status and ethnicity. In total, seroprevalence data from 928 individuals, all from London, was available.

**Results:**

Overall, no difference between HPV seroprevalence by immune status was observed (P = 0.3) among Caucasian or among non-Caucasian individuals, with seroprevalence varying from 87% to 94% across different immune status and ethnic groups. Those individuals seropositive to multiple types of one genus were more likely to be seroreactive to multiple types of another genus, independent of immune status or ethnicity. Lower seroprevalence for gammaHPV 4, and to a lesser extent gammaHPV 48, were observed among OTR compared to IC and dialysis patients. Higher seroprevalence against antibodies to betaHPV 93 were detected more frequently in non-Caucasians than Caucasians whereas muHPV 1 and, to a lesser extent, gammaHPV 4 were found more frequently among Caucasians - these findings were independent of immune status. Within non-Caucasian subgroups, the seroprevalence of 8 HPV (alpha-mucosal HPV16 and 13, alpha-cutaneous HPV7 and 2, betaHPV8, 17, 23 and 38) was significantly (P < 0.02) higher in Black compared to Asian patients. HPV16 being sexually transmitted, this might suggest a potential sexual route of transmission for some beta HPV types.

**Conclusion:**

We did not observe major disturbance in antibody response between immunocompetent, dialysis and OTR individuals, but significant differences in HPV seroprevalence were identified according to ethnicity. Further research is needed to clarify the natural history of cutaneous HPV, particularly given the growing research interest in its possible role in the pathogenesis of cutaneous SCC.

## Background

Genital human papillomaviruses (HPV6 and HPV11) are the most common sexually transmitted infectious agents in the UK [1]. Associations include younger age, lifetime number of sexual partners, socio economic status and ethnicity (black>white>asian) [2]. A higher seroprevalence of mucosal type HPV16, an important cause of cervical cancer, has also been associated with these factors and, additionally to female sex, to men who have sex with men and to urban location [3]. The natural history and risk factors associated with mucosal HPV infection have been studied intensively [4] but the natural history of cutaneous HPV is less clear [5].

Here, we investigate the seroprevalence for 34 HPV types detected using Luminex technology among people in London with different immune status and ethnicity. Information on 409 OTR without skin cancer (243 Caucasian and 166 non-Caucasian) and 152 immunocompetent (IC) individuals (102 Caucasians and 50 non-Caucasians) were included. Patients with end-stage renal disease on dialysis (222 Caucasians and 145 non-Caucasians), at increased risk of infections and cancers [6] probably due to abnormalities of the immune functions [7,8], were also included.

## Methods

### Study population

The present study was conducted as part of research examining the relationship between antibodies against HPV-L1 antigens for 34 HPV types and cutaneous squamous cell carcinoma among OTR. Further details of the study methods can be found elsewhere [9]. Briefly, a nested case-control study was conducted in a cohort of transplant recipients from Barts and London NHS Trust, recruited between October 2002 and August 2006. In London, all patients have access to a dedicated dermatology clinic following their usual visit to the transplant centre and undergo routine skin examinations, at which all benign and malignant lesions are recorder and treated if necessary. A trained nurse interviewed participants using a standard questionnaire and obtained demographic details, smoking and alcohol history, medical history, self-reported ultraviolet (UV) radiation exposure, history of viral infection, transplantation details, gynaecological and reproductive history and questions on crowding throughout life. A blood sample was taken and serum, buffy coat and red blood cells were separated, aliquoted and frozen at -80°Celsius.

In order to assess whether the seroprevalence of HPV was affected by immune status, IC patients were also recruited, and included Caucasians and non-Caucasians without a history of skin cancer enrolled from ophthalmology, plastic surgery or phlebotomy departments. A short questionnaire on basic socio-demographic details (sex, date of birth and ethnicity) and skin cancer history was completed and a blood sample was obtained. In order to evaluate the influence of renal failure pre-transplantation on HPV seroprevalence, stored sera from Caucasian and non-Caucasian dialysis patients with no history of transplantation, were also included. Skin cancer history was not available for these patients, although it was known that none had previously attended the dermatology department for treatment of skin cancer. Basic socio-demographic details (sex, date of birth and ethnicity) were provided from the hospital renal database.

In total, information on 409 OTR without skin cancer (243 Caucasian and 166 non-Caucasian of whom 5 patients [1%] with a solid organ graft other than kidney), 152 immunocompetent (IC) individuals (102 Caucasians and 50 non-Caucasians) and 367 patients with end-stage renal disease on dialysis (222 Caucasians and 145 non-Caucasians) were included.

### Ethical approval

The study has been approved by the East London and City Health Authority Research Ethics Committee.

### HPV multiplex serology

HPV antibody detection was performed by multiplex serology, an antibody detection method that is based on a glutathione *S*-transferase (GST) capture enzyme-linked immunosorbent assay, as previously described [10,11] in combination with fluorescent bead technology [12,13]. All antigens were expressed in *E. coli *as double fusion of full-length viral proteins with a N-terminal GST domain and a C-terminal peptide consisting of the last 11 amino acids from the large T antigen of simian virus 40 [10]. The expression constructs for the full length L1 proteins of all HPV types analyzed here (mucosal alpha: 6, 13 and 16; cutaneous alpha: 2, 3, 7 and 27; beta: 5, 8, 9, 15, 17, 20, 23, 24, 36, 38, 49, 75, 76, 92, 93, 96; gamma: 4, 65, 95, 48, 50, 60; nu: 41; mu: 1, other types: 101 and 103) are described in detail elsewhere [5,11,14]. Glutathione-casein was coupled to internally fluorescence-labeled polystyrene beads (Luminex, Austin, TX), and fusion proteins were affinity-purified on the beads directly in a one-step procedure. Beads with GST and the C-terminal peptide alone were prepared for background determination. Binding of the antigens (i.e. the GST fusion proteins) to various bead sets was verified with a monoclonal antibody against the common C-terminal peptide [10]. The differently labelled bead sets carrying different antigens were mixed and incubated in 96-well plates with human plasma diluted 1:100 in blocking buffer, as described previously [13]. The analyses were performed blinded with respect to the immune status of the samples. Antibodies bound to the beads via the viral antigens were then stained with biotinylated anti-human immunoglobulin and fluorescent reporter conjugate streptavidin-R-phycoerythrin. Antibodies bound to antigens on beads were quantified via the reporter fluorescence in the Luminex analyzer, which also identified the internal bead colour and thus the antigen carried by the bead. Antibody quantity was determined as the median R-phycoerythrin fluorescence intensity (MFI) from at least 100 beads of the same internal colour after subtraction of background reactivity (GST and C-terminal peptide alone). The assay reproducibility was high (R^2 ^= 0.97) [12,15]. More information on quality control has been described elswewhere [15]. For all HPV types but HPV6 analyzed here, MFI cut-offs to define seropositivity for all antigens were set to 200 MFI as described and discussed previously [5,14]. To reduce the influence of borderline seropositive sera, a stringent (doubled) cut-off of 400 MFI was applied to HPV6. In our previous analysis [14], data analysis using geometric mean MFI values instead of cut-off values did not materially change the results.

### Statistical methods

To assess the relationship between seropositivity to a single HPV type and ethnicity or immune status, logistic regression adjusted for sex, age at recruitment (<45, 45-59, ≥60 years) and, if appropriate, time since transplantation (<5, 5 to 9, ≥10 years) was applied. Where results are presented in the form of plots, black squares indicate the point estimates and horizontal lines represent 95% confidence intervals (CI). The area of the square is proportional to the amount of statistical information available (inverse of the variance of the logarithm of the estimate). To examine the association between multiple HPV seropositivity and ethnicity or immune status, negative binomial regression adjusted for the same factors was used since over-dispersion was observed when Poisson models were fitted (likelihood ratio test for the null hypothesis of no overdispersion was rejected with P < 0.001). Linear regression was used to compare the means between several groups. Skin type of non-Caucasians was defined using Fitzpatrick classification scale as follows (V) Asian, Middle Eastern and (VI) African/Afro-Caribbean. Detailed ethnicity information was only available for OTR and was defined as Caucasian (those who identified themselves as 'White' and were usually individuals of European descent) and non-Caucasian (those who identified themselves as 'Asian', 'Far Eastern', 'Black' or other' - usually individuals of non-European descent). Associations between age at recruitment, sex and HPV seropositivity was examined by immune status using logistic regression among Caucasian patients.

Sensitivity analysis was performed to compare HPV seroprevalence among IC, dialysis and transplant patients with kidney graft only. To deal with multiple significant tests the level of statistical significance was set to 1% and when a sufficient number of patients was available, agreement of results across population was used to detect genuine associations. Missing value categories were added to adjustment variables with incomplete information in order to retain all the observations in the analyses. Likelihood ratio tests were used to assess heterogeneity tests. All P-values are two-sided. Statistical analyses were carried out using STATA 9 (StataCorp, 2005).

## Results

### Participants

Table 1 shows the distribution of sex, time since transplantation and age at recruitment by ethnicity and immune status. In total, 243 Caucasian and 166 non-Caucasian OTR (5 patients [1%] with a solid organ graft other than kidney) without skin cancer, 102 Caucasian and 50 non-Caucasian IC patients without skin cancer, and 222 Caucasian and 145 non-Caucasian dialysis patients were available for analysis. Overall, seroprevalence data from 928 individuals were therefore available. Among Caucasian patients, OTR were on average younger than IC (P = 0.003) and dialysis patients (P < 0.001) but there was no statistical difference between the distribution of males and females in the 3 groups. Among non-Caucasian patients, the proportion of women was higher among immunocompetent individuals than OTR or dialysis patients and, IC individuals were also on average younger than the 2 other groups (P-heterogeneity = 0.01). Caucasian patients tended to have been transplanted for longer than non-Caucasians (mean [SD] in years: 9.6 [7.1] versus 7.0 [6.4]; P = 0.0002).

**Table 1:** Descriptive statistics for age at recruitment, sex and time since transplantation by immune status and ethnicity

	**CAUCASIANS**			**NON-CAUCASIANS**		
		
**Immune status Number**	**OTR**	**IC**	**dialysis**	**OTR**	**IC**	**dialysis**
		
	**N = 243**	**N = 102**	**N = 222**	**N = 166**	**N = 50**	**N = 145**
**sex**						
Ratio (M/F)	1.6 (150/93)	1.0 (50/52)	1.5 (132/90)	1.4 (96/70)	0.6 (18/32)	1.3 (83/62)
**age at recruitment (yr)**			***P-Het. = 0.1***			***P-Het. = 0.01***
Mean [SD] range	47 [13] 21 to 83	53 [18] 23 to 91	54 [14] 19 to 85	47 [12] 20 to 78	41 [16] 17 to 73	48 [13] 18 to 80
**time since transplantation (yr)**			***P-value^1 ^< 0.001***			***P-value^1 ^= 0.01***
Mean [SD] range	9.6 [7.1] 1 mth to 29.0 yr	NA	NA	7.0 [6.4] 3 mths to 28.4 yr	NA	NA
				***P-value_Caucasian versus non Caucasian OTR_^1 ^= 0.0002***		

N: Total number; no: number; IC: immunocompetent; OTR: organ transplant patients; mth: month; yr: year; NA: not available;

M: male; F: female; Het.: P-value for heterogeneity using logistic regression model; ^1^: P-value for difference between means using linear regression model.

Of the 166 non-Caucasian transplant recipients, 54% identified themselves as Asian, 31% as Black, 8% as Far Eastern and 7% as other ethnic group. In terms of country of birth (excluding missing data for 8% of Caucasians and 33% of non-Caucasians), 96% of Caucasian OTR were born in Europe and 83% of non-Caucasians were born outside Europe (41% Indian subcontinent, 32% Africa, 11% Caribbean, 10% Far East and 6% Middle East).

### Overall HPV seroprevalence by ethnicity and immune status

**Figure 1:**
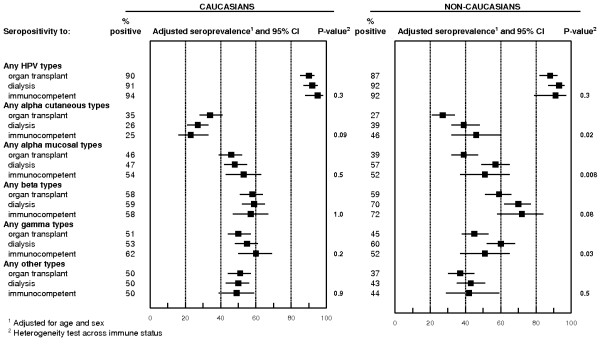
**Adjusted seroprevalence of HPV antibodies among Caucasian patients [243 organ transplant patients, 222 dialysis patients and 102 immunocompetent] and among non-Caucasian patients [166 organ transplant patients, 145 dialysis patients and 50 immunocompetent patients]**.

Figure 1 shows the overall seroprevalence for any HPV type and for each genus by ethnicity across immune status. Seroprevalence to at least 1 HPV type were 90%, 91% and 94% among Caucasians and 87%, 92% and 92% among non-Caucasian patients for OTR, dialysis and immunocompetent patients, respectively (both P-values for heterogeneity across immune status = 0.3). There was also no statistically significant difference by immune status among Caucasian patients whereas non-Caucasian transplant patients tended to have lower seroprevalence for any alpha cutaneous and mucosal, and gamma types than non-Caucasian dialysis patients. Seropositivity to cutaneous alpha types was also higher in non-Caucasian immunocompetent individuals (46%) compared to non-Caucasian OTR (27%).

### HPV seroprevalence and ethnicity

**Figure 2:**
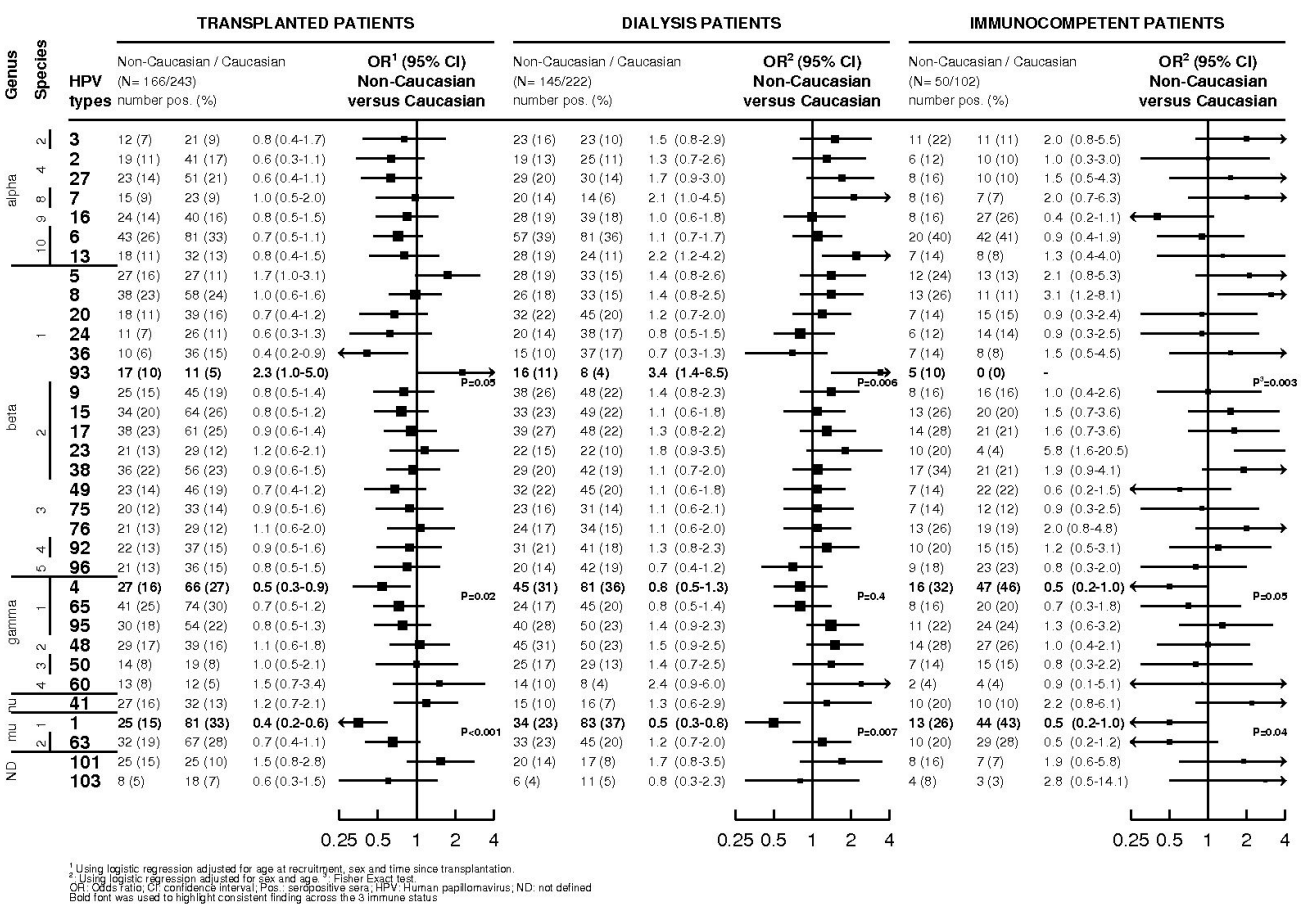
**Adjusted odds ratios of being seropositive to one human papillomavirus type by immune status and ethnicity**.

Figure 2 shows the odds ratios for being seropositive to each single HPV type in non-Caucasian versus Caucasian patients by immune status. Among transplant recipients, the prevalence of antibodies against betaHPV5 (Odd ratios [OR]: 1.7 and 95% confidence interval (CI): 1.0 to 3.1; P = 0.05), and betaHPV93 (OR: 2.3; 95% CI: 1.0 to 5.0; P = 0.05) were higher among non-Caucasian than Caucasian patients whereas for HPV 1 (OR: 0.4; 95% CI: (0.2 to 0.6), P < 0.001) and to a lesser extent gammaHPV4 (OR: 0.5; 95% CI: 0.3 to 0.9; P = 0.02) seroprevalence were lower in non-Caucasian patients. Differences identified among OTR in relation to ethnicity were corroborated for HPV93 (dialysis patients - OR: 3.4; 95% CI: 1.4 to 8.5; P = 0.006; IC - OR not available [0% in Caucasians versus 10% in non-Caucasians]; P-exact = 0.003) and HPV1 (dialysis patients - OR: 0.5; 95%CI: 0.3 to 0.8; P = 0.007; IC patients - OR: 0.5; 95% CI: 0.2 to 1.0; P = 0.04) and to lesser extent for HPV4 (dialysis patients - OR: 0.8; 95%CI: 0.5 to 1.3; P = 0.4; IC patients - OR: 0.5; 95% CI: 0.2 to 1.0; P = 0.05), but not for HPV5 or other HPV types among dialysis and IC patients. Exclusion of non-renal transplant patients (n = 5) did not materially change results (data not shown). There was no difference at the 1% level of significance between Caucasian and non-Caucasian OTR regarding multiple HPV seropositivity for any types, any alpha, cutaneous alpha, mucosal alpha, beta, gamma or other types (nu, mu and 2 not defined types) (data not shown).

Table 2 shows the seroprevalence for each HPV type within non-Caucasian OTR patients for the 2 main ethnic groups (Black [Africa/Afro-Caribbean] versus Asian). Black patients were more likely to be seroreactive to mucosal HPV16 (P = 0.01) and HPV13 (P = 0.02), and to cutaneous HPV7 (P = 0.007) and HPV2 (P = 0.03). Higher seropositivity for betaHPV 17, 23 and 38 (species 2) and to betaHPV 5 (species 1) was also observed in Black individuals compared to Asians. Results on multiple seropositivity between the 90 Asian and the 51 Black OTR reflected those findings of single seropositivity with Black patients more likely to be seroreactive to multiple alpha cutaneous HPV types (count ratio [CR]: 1.6; 95%CI: 1.0 to 2.7; P = 0.05), to multiple mucosal HPV types (CR: 2.0; 95%CI: 1.1 to 3.7; P = 0.02) and to betaHPV of species 2 (CR: 2.4; 95% CI: 1.3 to 4.5; P = 0.006) (data not shown).

**Table 2:** Seroprevalence for single HPV types among organ transplant patients for the 2 main ethnic groups of non-Caucasian patients

			**TWO MAIN ETHNIC GROUPS AMONG NON-CAUCASIAN PATIENTS**			
			
			**Asian** **N = 90**	**Black** **N = 51**		
			
**age (mean [SD])**			**46 ** [13]	**47 **[12]		
**ratio sex (M/F)**			**1.4 (53/37)**	**1.2 (28/23)**		
**Mean time since transplantation [SD]**			**7.9 (6.6)**	**4.7 (5.1)**		
**genus**	**species**	**types**	**POS (%)**	**POS (%)**	**OR (95%CI)**	***P-het.*^1^**

**alpha**	2	3	7 (8)	4 (8)	0.9 (0.2-3.3)	**0.8**
	4	2	6 (7)	10 (20)	3.6 (1.2-11.3)	**0.03**
	4	27	10 (11)	9 (18)	1.8 (0.6-4.9)	**0.3**
	8	7	2 (2)	9 (18)	9.5 (1.8-49.3)	**0.007**
	9	16	9 (10)	13 (25)	3.7 (1.4-10.0)	**0.01**
	10	6	23 (26)	11 (22)	0.9 (0.4-2.1)	**0.8**
	10	13	5 (6)	9 (18)	4.5 (1.3-15.6)	**0.02**

**beta**	1	5	11 (12)	12 (24)	2.9 (1.1-7.5)	**0.03**
	1	8	20 (22)	11 (22)	1.0 (0.4-2.4)	**1.0**
	1	20	8 (9)	7 (14)	1.5 (0.5-4.6)	**0.5**
	1	24	5 (6)	4 (8)	1.5 (0.4-6.1)	**0.6**
	1	36	4 (4)	4 (8)	1.9 (0.4-8.3)	**0.4**
	1	93	5 (6)	8 (16)	3.0 (0.9-10.2)	**0.1**
	2	9	10 (11)	11 (22)	2.4 (0.9-6.5)	**0.1**
	2	15	16 (18)	12 (24)	1.5 (0.6-3.6)	**0.4**
	2	17	12 (13)	18 (35)	4.4 (1.8-11.0)	**0.001**
	2	23	7 (8)	11 (22)	3.7 (1.2-11.2)	**0.02**
	2	38	13 (14)	17 (33)	3.3 (1.4-8.0)	**0.007**
	3	49	13 (14)	7 (14)	0.9 (0.3-2.4)	**0.8**
	3	75	8 (9)	8 (16)	2.0 (0.7-6.0)	**0.2**
	3	76	8 (9)	10 (20)	2.4 (0.9-6.8)	**0.1**
	4	92	8 (9)	8 (16)	1.9 (0.6-5.7)	**0.2**
	5	96	11 (12)	7 (14)	1.2 (0.4-3.4)	**0.8**

**gamma**	1	4	14 (16)	7 (14)	0.7 (0.3-2.1)	**0.6**
	1	65	23 (26)	11 (22)	0.6 (0.3-1.5)	**0.3**
	2	95	16 (18)	9 (18)	1.0 (0.4-2.6)	**0.9**
	2	48	14 (16)	9 (18)	1.1 (0.4-2.8)	**0.9**
	3	50	10 (11)	4 (8)	0.9 (0.2-3.4)	**0.9**
	4	60	8 (9)	4 (8)	1.0 (0.2-4.0)	**1.0**

**nu**		41	6 (7)	9 (18)	1.1 (0.4-2.8)	**0.9**

**mu**	1	1	14 (16)	6 (12)	0.7 (0.3-2.1)	**0.6**
	2	63	15 (17)	12 (24)	1.5 (0.6-3.7)	**0.4**

**ND**		101	13 (14)	9 (18)	1.2 (0.5-3.2)	**0.7**
		103	2 (2)	4 (8)	3.1 (0.5-18.5)	**0.2**

POS: number seropositive patients; N: number; ND: Not defined; Het.: heterogeneity; SD: standard deviation;

M: Male; F: Female. ^1 ^P-values were calculated using logistic regression adjusted for age at recruitment, time

since transplantation and sex.

### HPV seroprevalence by immune status

In Table 3, seroprevalence of the 34 HPV types were examined, with comparison between OTR, IC and dialysis patients by ethnic group. No association was found between HPV seroprevalence and sex or age within each immune status group (data not shown). As expected higher antibody response was observed among women and younger patients for mucosal types, but these results did not reach statistical significance at the 1% level (data not shown). Those individuals seropositive to multiple types of one genus were more likely to be seroreactive to multiple types of another genus independently of ethnicity or immune status (data not shown). The overall mean number of HPV types identified in people was similar between the three immune groups (6 types [SD: 6], 6 [SD: 7] and 6 [SD: 7] among Caucasian IC, OTR and dialysis patients and 7 types [SD: 8], 5 [SD: 7] and 6 [7] among non-Caucasian IC, OTR and dialysis patients).

**Table 3:** Seroprevalence for single HPV types by immune status (transplant, dialysis and immunocompetent individuals)

**Ethnicity**			**Caucasians**				**Non-Caucasians**			
		
**number**			**102**	**222**	**243**		**50**	**145**	**166**	
**immune status**			**IC**	**dialysis**	**OTR**		**IC**	**Dialysis**	**OTR**	
**genus**	**species**	**types**	**% POS**	**% POS**	**% POS**	**P-het^1^**	**% POS**	**% POS**	**% POS**	**P-het^1^**
	
**alpha**	2	3	11	10	9	**0.7**	22	16	7	**0.008**
	4	2	10	11	17	**0.07**	12	13	11	**0.9**
	4	27	10	14	21	**0.02**	16	20	14	**0.4**
	8	7	7	6	9	**0.5**	16	14	9	**0.3**
	9	16	26	18	16	**0.1**	16	19	14	**0.4**
	10	6	41	36	33	**0.4**	40	39	26	**0.02**
	10	13	8	11	13	**0.2**	14	19	11	**0.1**

**beta**	1	5	13	15	11	**0.6**	24	19	16	**0.6**
	1	8	11	15	24	**0.008**	26	18	23	**0.3**
	1	20	15	20	16	**0.3**	14	22	11	**0.03**
	1	24	14	17	11	**0.1**	12	14	7	**0.1**
	1	36	8	17	15	**0.09**	14	10	6	**0.2**
	1	93	0	4	5	**-**	10	11	10	**1.0**
	2	9	16	22	19	**0.4**	16	26	15	**0.05**
	2	15	20	22	26	**0.5**	26	23	20	**0.7**
	2	17	21	22	25	**0.7**	28	27	23	**0.7**
	2	23	4	10	12	**0.03**	20	15	13	**0.3**
	2	38	21	19	23	**0.7**	34	20	22	**0.1**
	3	49	22	20	19	**0.7**	14	22	14	**0.1**
	3	75	12	14	14	**0.9**	14	16	12	**0.6**
	3	76	19	15	12	**0.4**	26	17	13	**0.1**
	4	92	15	18	15	**0.7**	20	21	13	**0.1**
	5	96	23	19	15	**0.3**	18	14	13	**0.8**

**gamma**	1	4	46	36	27	**0.001**	32	31	16	**0.004**
	1	65	20	20	30	**0.03**	16	17	25	**0.1**
	1	95	24	23	22	**0.9**	22	28	18	**0.1**
	2	48	26	23	16	**0.05**	28	31	17	**0.01**
	3	50	15	13	8	**0.1**	14	17	8	**0.05**
	4	60	4	4	5	**0.9**	4	10	8	**0.4**

**nu**		41	10	7	13	**0.2**	20	10	16	**0.1**

**mu**	1	1	43	37	33	**0.2**	26	23	15	**0.09**
	2	63	28	20	28	**0.3**	20	23	19	**0.7**

**ND**		101	7	8	10	**0.6**	16	14	15	**0.9**
		103	3	5	7	**0.3**	8	4	5	**0.7**

IC: Immunocompetent; OTR: Organ transplant patients; ND: Not defined; % POS: percentage of seropositive patients.

^1 ^Heterogeneity tests using logistic regression between immunocompetent, dialysis and transplant Caucasian patients

from London, adjusted for age and sex.

The only consistent finding across ethnicity groups was lower HPV4 seroprevalence and, to a lesser extent, gammaHPV 48 among OTR compared to IC or dialysis patients. Among Caucasian patients, seroprevalence of antibodies against betaHPV8 was 24% in OTR compared to 15% among dialysis and 11% among immunocompetent individuals (P-value for heterogeneity = 0.008). Otherwise, HPV seroprevalence differed little for most types across the different immune status groups, after adjustment for age and sex. For multiple HPV seropositivity, there was also no statistical difference at the 1% level of significance between the three immune status groups among Caucasian patients whereas seropositivity to multiple mucosal alpha HPV types tended to be higher among dialysis and immunocompetent patients compared to OTR (data not shown).

## Discussion

Little is known of the seroepidemiology of HPV, with the exception of those mucosal types associated with cancer of the uterine cervix [3] or with genital warts [2]. We report here on the seroprevalence and risk factors for 34 HPV types detected using Luminex technology among 243 Caucasian and 166 non-Caucasian OTR without skin cancer, 367 individuals with end stage renal failure on dialysis (222 Caucasians and 145 non-Caucasians) and 152 IC individuals (102 Caucasians and 50 non-Caucasians), in order to compare the HPV seroprevalence in patients with differing immune status and ethnicity. The aim was to understand better the humoral response of cutaneous HPV types.

As expected [16], mucosal and cutaneous HPV were ubiquitous with more than 87% of OTR, IC and dialysis patients, of Caucasian and non-Caucasian origin, being seroreactive to one or more types. Susceptibility to multiple HPV infections did not seem to be directly related to immunosuppressive treatments, since IC and dialysis patients that were found to be seropositive to multiple HPV types of one genus were also more likely to be seroreactive to multiple types of another. It has to be borne in mind that the groups examined differed in age and sex distributions, however we did not observe any association with age and sex in relation to cutaneous HPV types within ethnic groups and immune status groups.

To our knowledge, no previously reported studies have investigated the association between the prevalence of cutaneous HPV antibodies by ethnicity. Consistently across immune status, we observed statistically significant differences between Caucasians and non-Caucasians: betaHPV 93 was higher in non-Caucasians, whereas muHPV type 1 and to a lesser extent HPV4 seroprevalence were lower. As most Caucasians were born in Europe and non-Caucasian outside Europe we were not able to distinguish if the observed differences were confounded by birth country. There were some limitations to our analyses on ethnicity as diverse groups (Asian, Black [African and Afro-Caribbean], Far Eastern or other) with different birth country were all pooled in the non-Caucasian category. Geographical studies are essential to examine further the HPV seroprevalence among different ethnicities. Studies on sexually transmitted diseases such as gonorrhoea and chlamydia based in London [17,18], Birmingham [19] or Leeds [2] have reported higher rates in Black compared to Asian populations. The higher seropositivity observed for mucosal alphaHPV 16 among Black patients compared to Asian individuals could support these previous reports. However, we did not find any significant difference in HPV6 - with associated genital warts - seroprevalence between Asian and Black. Interestingly, the only other clear association was the higher seroprevalence for beta species 2 observed among Black individuals compared to Asians which might suggest a similar mode of transmission. In another study, we found that, among Caucasian transplant patients without skin cancer, women with a self-reported history of abnormal smear tests were also more likely to have higher seroprevalence for alpha mucosal and beta types, compared to those without such history (Casabonne *et al*., this issue), again suggesting a possible sexual route of transmission for some beta types.

In relation to immune status, we observed that seroprevalence of most HPV types did not differ substantially, after controlling for age and sex, between Caucasian IC individuals, dialysis patients without a history of transplantation and OTR, suggesting apparently low disturbance in production of antibodies by immunological status. The only notable exception was a lower HPV4 seroprevalence among OTR compared to dialysis or IC patients independent of ethnicity. Unfortunately, we do not have complete information on the type of dialysis (continuous ambulatory peritoneal dialysis or haemodialysis) to explore whether this specifically affected seroprevalence. The observed differences in overall seroprevalence by genera among non-Caucasian patients might be due to the pooling of very heterogeneous groups of non-Caucasians [18].

## Conclusion

In summary, we did not find major differences in the prevalence of antibodies against 34 HPV types among people with different immune states. Ethnicity did show significance differences, but results should be interpreted with caution since ethnic groups with different birth country, cultural and socioeconomic backgrounds were pooled together. Further research is needed to clarify the risk factors and the natural history of these viruses.

## List of Abbreviations

HPV: human papilloma virus; OTR: organ transplant recipients; IC: Immunocompetent; OR: odds ratio; CI: confidence interval.

## Competing interests

The authors declare that they have no competing interests.

## Authors' contributions

CP, CH and RN conceived the study. LM, TW and DC participated in acquisition of all the biological material and data. MP, TW and KM developed the HPV assays and KM analysed the samples. DC analysed the data and drafted the manuscript. All authors read, contributed to and approved the manuscript.
